# Integrated Multi-Omic Analysis of Paired Primary and Peritoneal Metastatic Tumors Reveals GPAA1 as a Therapeutic Target in Colorectal Cancer

**DOI:** 10.3390/ijms27209106

**Published:** 2026-10-14

**Authors:** Zhiwei Zhang, Zhiyuan Gong, Akao Zhu, Cao Chen, Yeting Hu, Jian Wang, Lifeng Sun

**Affiliations:** 1 Department of Colorectal Surgery and Oncology, Key Laboratory of Cancer Prevention and Intervention, Ministry of Education, The Second Affiliated Hospital, Zhejiang University School of Medicine, Hangzhou 310003, China; 2 Department of Cancer Institute, The Second Affiliated Hospital, Zhejiang University School of Medicine, Hangzhou 310003, China

**Keywords:** colorectal cancer, peritoneal metastasis, GPAA1, multi-omics, epithelial-mesenchymal transition

## Abstract

Peritoneal metastasis confers a dismal prognosis in colorectal cancer (CRC-PM), with median survival rarely exceeding 20 months. Here we employed multi-omic profiling to identify actionable drivers of CRC-PM. We performed whole-exome sequencing (WES) and label-free quantitative proteomics on 13 and 7 matched primary-peritoneal metastasis pairs, respectively. Public single-cell (GSE183916 and GSE132465) and spatial transcriptomic datasets (GSE225857) were integrated to dissect cellular heterogeneity. TCGA cohorts were used for survival analysis, and functional validation was performed in DLD1 cells. GPAA1, a catalytic subunit of the GPI transamidase complex, emerged as the most consistently upregulated protein in peritoneal metastases across discovery and validation proteomic cohorts. High GPAA1 expression correlated with shorter recurrence-free survival (HR = 1.72, *p* = 3.4 × 10^−5^). Single-cell analysis localized GPAA1 to malignant cells exhibiting oxidative phosphorylation and enhanced stromal crosstalk. GPAA1 overexpression promoted proliferation, migration, and EMT via AKT activation, whereas knockdown reversed these phenotypes. Our findings identify GPAA1 as a candidate driver of peritoneal metastasis in CRC, linking GPI anchor biosynthesis to metabolic reprogramming and AKT-mediated EMT. GPAA1 may serve as a prognostic marker in colorectal cancer.

## 1. Introduction

Colorectal cancer (CRC) is the third most commonly diagnosed malignancy and the second leading cause of cancer-related mortality worldwide [1,2]. Peritoneal dissemination occurs in 10–15% of patients at diagnosis and develops in another 20–25% during follow-up [3], and portends a median survival of less than 20 months despite modern systemic therapy [4]. The molecular programs specific to peritoneal tropism remain largely uncharacterized, and no validated biomarkers exist to identify patients at high risk [5].

Peritoneal colonization proceeds through a multistep cascade—detachment, intraperitoneal survival, mesothelial adhesion, and stromal invasion—each requiring distinct molecular adaptations [6,7,8,9]. Whereas liver and lung metastases have been extensively profiled, the molecular landscape specific to peritoneal dissemination remains understudied [10].

Integrative multi-omic profiling can reveal metastatic drivers that evade single-platform detection [11,12]. In CRC peritoneal metastasis, such approaches may uncover context-specific dependencies with therapeutic potential [13].

Glycosylphosphatidylinositol (GPI) anchor biosynthesis is a conserved post-translational modification pathway that tethers proteins to the cell membrane, regulating cell surface signaling, adhesion, and immune recognition [14]. The GPI transamidase complex, comprising GPAA1, PIGT, PIGU, and PIGS, catalyzes the transfer of GPI anchors to target proteins [15]. Dysregulation of individual GPI-anchored proteins—including CEA, CA125, and mesothelin—has been linked to cancer progression [16], but whether the GPI biosynthetic machinery itself drives metastasis is unclear [17]. A recent study reported that GPAA1 promotes gastric cancer progression via ERBB signaling [18]. Independently, Zhu et al. (2025) employed integrative multi-omics analysis and experimental validation to demonstrate that GPAA1 mediates a cold-tumor phenotype and immune evasion in colorectal cancer [19]. By contrast, our study is specifically designed around paired primary and peritoneal metastatic tumors and uses quantitative proteomics to resolve protein-level GPAA1 upregulation in metastatic lesions. However, whether GPAA1 specifically drives peritoneal metastasis in CRC—rather than primary tumor progression—and the underlying multi-omic programs remain undefined.

The aim of this study was to systematically identify proteins that are selectively dysregulated in peritoneal metastases compared to paired primary tumors. In the functional experiments involving GPAA1, we evaluated the hypothesis that GPAA1 facilitates peritoneal colonization by promoting proliferation, migration, and epithelial–mesenchymal transition via AKT signaling pathways. Here we report an integrated multi-omic analysis of paired primary and peritoneal metastatic CRC samples, combining WES, quantitative proteomics, single-cell transcriptomics, and spatial transcriptomics. We identify GPAA1 as a reproducibly upregulated protein in peritoneal metastases and demonstrate its prognostic value, association with metabolic reprogramming and tumor–stromal crosstalk, and functional role in promoting proliferation, migration, and EMT via AKT activation. Our findings establish GPAA1 as a candidate biomarker and therapeutic target in CRC peritoneal metastasis.

## 2. Results

### 2.1. Genomic and Proteomic Landscape of Paired Primary and Peritoneal Metastatic Colorectal Cancer

To systematically characterize the molecular alterations driving colorectal cancer (CRC) peritoneal metastasis, we performed whole-exome sequencing (WES) on 13 pairs of matched primary tumors and peritoneal metastases, alongside label-free quantitative proteomics on 7 pairs of these samples (Figure 1A). The mutational landscape revealed a comparable tumor mutational burden (TMB) between primary and metastatic lesions, with frequent mutations in APC, KRAS, TP53, and TTN observed in both groups (Figure 1B,C). It is noteworthy that FAT4 and MUC16 exhibited increased mutation frequencies in peritoneal metastases compared to primary tumors (Figure 1D,E). Nonetheless, due to the limited sample size, the substantial size of these genes, and the lack of a paired analysis with correction for multiple testing, these results are preliminary. KEGG pathway enrichment analysis of the proteomic data highlighted significant dysregulation in cadherin signaling, cytoskeleton organization, and cell adhesion molecule interactions in metastatic lesions (Figure 1F). However, there are no significant copy-number alterations or recurrent mutations in GPAA1. This suggests that GPAA1 upregulation in peritoneal metastases is unlikely driven by genomic amplification, prompting us to investigate protein-level dysregulation as an alternative mechanism.

### 2.2. Proteomic Profiling Identifies GPAA1 as a Prominently Up-Regulated Protein in Peritoneal Metastasis

To identify differentially expressed proteins driving colorectal cancer peritoneal metastasis, we performed proteome-wide paired comparison between matched primary and peritoneal metastatic samples. UMAP clustering clearly segregated primary tumors from peritoneal metastases, with paired samples from individual patients connected by dashed lines, indicating distinct proteomic profiles upon metastatic dissemination (Figure 2A). Differential protein analysis (paired limma, *p* < 0.05) revealed a robust set of up-regulated proteins in peritoneal metastases, among which GPAA1 emerged as one of the most significantly elevated proteins, alongside MEST, RBM28, and WDR74 (Figure 2B).

To validate these findings, we analyzed an independent cohort of primary and peritoneal metastatic samples from 392 patients (Table 1). UMAP clustering again demonstrated clear separation between the two groups (Figure 2C), and differential protein analysis (independent samples, *p* < 0.05) confirmed GPAA1 as a consistently up-regulated protein in peritoneal metastases, together with ASC3, CMGA, and NAAE2 (Figure 2D). Venn diagram analysis of discovery and validation cohorts identified 28 proteins commonly up-regulated in both datasets, with GPAA1 present in the overlapping up-regulated set (Figure 2E).

KEGG pathway enrichment analysis of the up-regulated proteins highlighted significant activation of cytosolic DNA-sensing pathway, PPAR signaling, RNA degradation, nucleocytoplasmic transport, and cell cycle regulation in peritoneal metastases (Figure 2F). We applied Benjamini–Hochberg (FDR) correction to the paired differential proteomics results. However, owing to the small discovery cohort (7 paired samples), no proteins remained statistically significant after correction, and the reported findings therefore rely on nominal *p* values. Quantitative analysis of GPAA1 protein levels demonstrated significantly higher expression in peritoneal metastases compared to primary tumors in both the discovery cohort (Wilcoxon test, *p* = 0.0017) and the validation cohort (Wilcoxon test, *p* = 0.0425) (Figure 2G,H). Thus, GPAA1 is reproducibly upregulated at the protein level in CRC peritoneal metastases across two independent cohorts, prompting us to investigate its functional role.

### 2.3. Clinical Prognostic Value and Pathway Association of GPAA1 in Colorectal Cancer

To evaluate the clinical relevance of GPAA1 in colorectal cancer, we analyzed GPAA1 mRNA expression using publicly available transcriptomic datasets. GPAA1 expression was significantly up-regulated in tumor tissues compared to adjacent normal tissues (*p* < 0.001) (Figure 3A). Furthermore, GPAA1 expression was significantly higher in metastatic (stage IV) tumors than in non-metastatic (stage I-III) tumors (*p* = 0.0355), indicating a positive correlation between GPAA1 expression and metastatic progression (Figure 3B).

To investigate the biological pathways associated with GPAA1, we stratified colorectal cancer patients into GPAA1-high and GPAA1-low expression groups based on median expression. Differential gene expression analysis identified 179 up-regulated and 153 down-regulated genes in the GPAA1-high group (Figure 3C). Kaplan–Meier survival analysis revealed that high GPAA1 expression was significantly associated with shorter recurrence-free survival (RFS) (HR = 1.72, 95% CI: 1.33–2.23, log-rank *p* = 3.4 × 10^−5^) (Figure 3D). However, GPAA1 expression showed no significant association with overall survival (OS) (HR = 1.12, 95% CI: 0.92–1.37, log-rank *p* = 0.27) (Figure 3E). The lack of OS association may reflect confounding by subsequent treatment regimens in these publicly available cohorts.

We further performed univariable and multivariable Cox regression in the TCGA-COAD cohort to examine whether GPAA1 is an independent prognostic factor for disease-specific survival (DSS). GPAA1-high tumors showed a trend toward shorter DSS in univariable analysis (HR = 1.74, 95% CI: 1.05–2.91, *p* = 0.033), and this association remained significant after adjusting for stage, age, and sex (multivariable HR = 1.83, 95% CI: 1.07–3.13, *p* = 0.027), indicating that GPAA1 is an independent prognostic factor for DSS (Figure S1).

KEGG pathway enrichment analysis of differentially expressed genes (DEGs) between GPAA1-high and GPAA1-low groups highlighted significant enrichment in neuroactive ligand-receptor interaction, linoleic acid metabolism, hormone signaling, and folate transport and metabolism (Figure 3F). Hallmark gene set enrichment analysis (GSEA) further revealed that GPAA1-high tumors were significantly enriched in DNA repair, E2F targets, adipogenesis, estrogen response, fatty acid metabolism, and cholesterol homeostasis pathways (Figure 3G). Thus, elevated GPAA1 expression correlates with metastatic progression, shorter RFS, and activation of metabolic and proliferative pathways in CRC [19].

### 2.4. Single-Cell Analysis Reveals GPAA1-Mediated Metabolic Reprogramming and Enhanced Tumor–Stromal Interactions in Malignant Cells

To dissect the cellular heterogeneity and GPAA1-associated functional states in the colorectal cancer peritoneal metastatic microenvironment, we performed single-cell RNA sequencing (scRNA-seq) analysis on the GSE183916 and GSE132465 datasets (peritoneal metastasis, *n* = 5 patients/samples; primary tumor, *n* = 5 patients/samples; 23,389 cells retained after quality control). UMAP clustering identified ten major cell types: malignant cells, enterocytes, goblet cells, fibroblasts, endothelial cells, T cells, B cells, plasma cells, myeloid cells, and mast cells (Figure 4A). Feature plot analysis revealed that GPAA1 was predominantly expressed in malignant cell clusters, with relatively low expression in stromal and immune cell populations (Figure 4B).

Violin plot comparison of GPAA1 expression across cell types and tissue origins demonstrated that GPAA1 was significantly up-regulated in malignant cells from peritoneal metastases compared to those from primary tumors (*** *p* < 0.001), while enterocytes also showed elevated GPAA1 in metastatic lesions (* *p* < 0.05) (Figure 4C). To investigate the biological consequences of GPAA1 elevation in malignant cells, we stratified malignant cells into GPAA1-high and GPAA1-low subgroups. KEGG pathway enrichment analysis revealed that GPAA1-high malignant cells were significantly enriched in oxidative phosphorylation, proteasome, protein processing in endoplasmic reticulum, and thermogenesis pathways (Figure 4D). Gene Ontology (GO) biological process analysis further confirmed enrichment in ribonucleoprotein complex biogenesis, RNA splicing, mitochondrial translation, and electron transport chain (Figure 4F).

UMAP visualization of malignant cells colored by tissue origin showed substantial intermingling of primary and metastatic cells, with GPAA1 expression prominently elevated in the metastatic malignant cell subset (Figure 4E). CellChat-mediated cell–cell communication analysis revealed distinct interaction patterns between GPAA1-high and GPAA1-low malignant cells with the tumor microenvironment. GPAA1-high malignant cells exhibited enhanced outgoing signaling to fibroblasts, B cells, T cells, and myeloid cells, suggesting active remodeling of the peritoneal metastatic niche (Figure 4G). Conversely, GPAA1-high malignant cells received stronger incoming signals from fibroblasts and endothelial cells, indicating a reciprocal activation loop within the metastatic microenvironment (Figure 4H). Thus, at single-cell resolution, GPAA1 marks a metabolically active malignant cell state characterized by augmented tumor–stromal communication.

### 2.5. Spatial Transcriptomics and IHC Validation Confirm GPAA1 Up-Regulation in Peritoneal Metastasis and Its Association with Malignant Cell Spatial Distribution

To validate the proteomic and single-cell findings at the tissue level, we performed immunohistochemical (IHC) staining of FAT4, MUC16, and GPAA1 on matched adjacent normal tissues, primary colon cancer tissues, and peritoneal metastatic tissues. IHC analysis revealed that FAT4 expression was predominantly localized to adjacent normal epithelium and was progressively lost in primary tumors and peritoneal metastases. In contrast, MUC16 and GPAA1 showed minimal expression in normal tissues, moderate expression in primary tumors, and markedly strong staining in peritoneal metastatic lesions, with GPAA1 exhibiting particularly intense cytoplasmic and membranous staining in metastatic tumor cells (Figure 5A).

To further investigate the spatial distribution of GPAA1-expressing malignant cells, we performed exploratory spatial transcriptomic analysis on a representative peritoneal metastatic tissue section (10x Visium). Spatial feature plots showed that EPCAM-positive malignant cells were broadly distributed across the tissue, while GPAA1 expression was concentrated in specific malignant cell clusters (Figure 5B). Cell-type annotation of the spatial transcriptomic data identified distinct regions enriched for GPAA1-negative malignant cells (GPAA1-Mali), GPAA1-positive malignant cells (GPAA1+Mali), B cells, myeloid cells, stromal cells, and T cells (Figure 5C).

Correlation analysis of cell-type spatial co-localization revealed significant positive correlations between GPAA1+Mali and stromal cells, as well as between GPAA1+Mali and myeloid cells, suggesting a preferential spatial proximity of GPAA1-high malignant cells to the tumor stroma and immune infiltrates (Figure 5D). Niche decomposition analysis (MISTy) demonstrated that the contribution of para-15 (long-range interaction, 15-cell radius) signaling was particularly prominent in GPAA1+Mali and stromal cell niches, indicating extensive long-range intercellular communication within the metastatic microenvironment (Figure 5E).

Intra-niche interaction analysis further revealed that GPAA1+Mali cells engaged in strong outgoing signaling to stromal cells and myeloid cells, while receiving incoming signals from T cells and B cells within the para-15 spatial niche (Figure 5F,G). Together, these spatial and IHC data from a representative sample independently confirm GPAA1 upregulation in peritoneal metastases and suggest a link to specific tumor–stromal and tumor-immune spatial patterns. Validation in additional spatial transcriptomic datasets will be needed to generalize these observations.

### 2.6. Establishment of GPAA1 Gain- and Loss-of-Function Models in CRC Cells

To establish an in vitro model for functional investigation of GPAA1, we first assessed endogenous GPAA1 protein expression across a panel of colorectal cancer cell lines and normal colonic epithelial cells. Western blot analysis revealed that GPAA1 was expressed at variable levels in all tested colorectal cancer cell lines (RKO, LOVO, DLD1, SW620, SW480, HT29, HCT116), with relatively lower expression in the normal colonic epithelial cell line NCM460. Notably, DLD1 cells exhibited moderate endogenous GPAA1 expression, making them suitable for both overexpression and knockdown experiments (Figure 6A).

To validate GPAA1 expression in clinical specimens, we performed Western blot analysis on matched tumor (T) and adjacent normal (N) tissues from four colorectal cancer patients. The levels of GPAA1 protein were consistently elevated in tumor tissues relative to adjacent normal tissues across all four patient pairs, thereby corroborating the up-regulation of GPAA1 in primary colorectal cancer (Figure 6B). It is important to note that this comparison is limited to four matched pairs of primary colorectal cancer and adjacent normal tissues and does not encompass peritoneal metastatic samples. Consequently, it cannot establish an association specific to peritoneal metastasis.

To functionally investigate the role of GPAA1 in colorectal cancer, we generated stable GPAA1-overexpressing DLD1 cells using the PLV3-GPAA1 lentiviral vector and stable GPAA1-knockdown DLD1 cells using two independent shRNA sequences (ShGPAA1-1 and ShGPAA1-2). Western blot confirmed successful GPAA1 overexpression in PLV3-GPAA1-transduced cells compared to vector control (Figure 6C), and efficient GPAA1 knockdown in both ShGPAA1-1 and ShGPAA1-2 cells compared to ShNT non-targeting control (Figure 6D). Quantitative RT-PCR further validated a ~4-fold increase in GPAA1 mRNA levels in overexpressing cells (**** *p* < 0.0001) and a significant reduction in GPAA1 mRNA in both knockdown lines (* *p* < 0.05) (Figure 6E,F). These validated cell models were used for subsequent functional studies.

### 2.7. GPAA1 Promotes Colorectal Cancer Cell Proliferation, Migration, and Epithelial–Mesenchymal Transition via AKT Pathway Activation

To functionally validate the oncogenic role of GPAA1 in colorectal cancer, we performed a series of in vitro assays using the established GPAA1-modulated DLD1 cell models. CCK-8 proliferation assay demonstrated that GPAA1 overexpression significantly enhanced cell proliferation compared to vector control (** *p* < 0.01), while GPAA1 knockdown with both ShGPAA1-1 and ShGPAA1-2 significantly inhibited proliferation (** *p* < 0.01) (Figure 7A,B).

Colony-formation assays further confirmed that GPAA1 overexpression increased clonogenic capacity, as evidenced by larger and more numerous colonies, whereas GPAA1 knockdown markedly reduced colony formation (Figure 7C,D). Wound-healing assays revealed that GPAA1-overexpressing cells exhibited accelerated wound closure at 24 h compared to control cells, indicating enhanced migratory ability (Figure 7E). Transwell migration assays corroborated these findings, showing significantly increased migrated cell numbers in the GPAA1-overexpression group (Figure 7F).

To investigate the molecular mechanism underlying GPAA1-mediated phenotypic changes, we examined epithelial–mesenchymal transition (EMT) markers and AKT signaling pathway components [20]. Western blot analysis showed that GPAA1 overexpression down-regulated E-cadherin (epithelial marker) and up-regulated N-cadherin and Vimentin (mesenchymal markers), accompanied by increased phosphorylation of AKT (p-AKT) without altering total AKT levels (Figure 7G). Conversely, GPAA1 knockdown restored E-cadherin expression, reduced N-cadherin and Vimentin levels, and decreased p-AKT phosphorylation (Figure 7H). These data indicate that GPAA1 promotes CRC cell proliferation, migration, and EMT in association with AKT phosphorylation. Whether AKT activation is a direct downstream mediator of GPAA1 requires further rescue experiments using AKT pathway modulators.

### 2.8. GPAA1 Enhances In Vivo Tumor Cell Proliferation and Peritoneal Metastasis

To validate the promoting effect of GPAA1 on the malignant phenotype of colorectal cancer in vivo, we inoculated DLD1-Vector and DLD1-GPAA1-OE cells subcutaneously and intraperitoneally into BALB/c nude mice to establish subcutaneous xenograft and peritoneal metastasis models, respectively. In the subcutaneous tumor model, tumor volumes were measured regularly from day 10 post-inoculation. The results showed that the tumor growth rate in the GPAA1-OE group was significantly faster than that in the Vector group, and by day 20, the tumor volume in the GPAA1-OE group was significantly larger than that in the Vector group (Figure 8B, *p* < 0.05). At the experimental endpoint, both the volume and weight of subcutaneous tumors in the GPAA1-OE group were significantly greater than those in the Vector group (Figure 8A,C, *p* < 0.05), indicating that GPAA1 overexpression markedly promotes the in vivo proliferative capacity of colorectal cancer cells.

In the peritoneal metastasis model, mice in the GPAA1-OE group exhibited numerous diffuse metastatic nodules within the abdominal cavity, widely distributed across the mesentery, peritoneum, and surfaces of intra-abdominal organs, whereas the number of peritoneal metastatic foci in the Vector group was markedly reduced (Figure 8D). After isolation and collection of all peritoneal metastatic nodules, the GPAA1-OE group showed substantially greater numbers and larger sizes of metastatic nodules compared with the Vector group (Figure 8E), and the total weight of peritoneal metastatic tumors in the GPAA1-OE group was significantly higher than that in the Vector group (Figure 8F, *p* < 0.001). These in vivo findings are consistent with the in vitro results from CCK-8, colony formation, wound healing, and Transwell assays (Figure 7), collectively demonstrating that GPAA1 overexpression enhances in vivo proliferation and peritoneal tumor burden. Because in vivo loss-of-function experiments were not performed in this model, whether endogenous GPAA1 is strictly required for peritoneal metastasis remains to be established.

Otherwise, our in vivo experiments demonstrate enhanced peritoneal tumor growth upon GPAA1 overexpression but do not directly assess metastatic site preference. Accordingly, our conclusions distinguish enhanced peritoneal growth from peritoneal tropism.

## 3. Discussion

Peritoneal metastasis in CRC carries a dismal prognosis and lacks targeted therapies [4,21]. Through integrative multi-omic profiling of paired primary and metastatic samples, we identified GPAA1 as a consistently upregulated protein in peritoneal metastases and demonstrated its functional role in promoting proliferation, migration, and EMT via AKT signaling.

GPAA1 functions as a catalytic subunit of the GPI transamidase complex, which mediates attachment of GPI anchors to substrate proteins—a modification critical for cell surface localization and signaling [14]. Our finding that GPAA1 is reproducibly upregulated at the protein level in peritoneal metastases suggests that enhanced GPI anchor biosynthesis may be a specific feature of this metastatic site. The consistency of GPAA1 upregulation across discovery and validation proteomic cohorts, independently confirmed by IHC, argues against a chance finding and supports a functional role. Compared with Zhu et al. (2025) [19], who linked GPAA1 to a cold-tumor phenotype and immune evasion in CRC, our findings overlap in associating GPAA1 with immune-related and metabolic pathways but additionally identify protein-level upregulation in peritoneal metastases and demonstrate enhanced peritoneal tumor growth.

Pathway analyses linked high GPAA1 expression to DNA repair, E2F targets, and metabolic reprogramming—hallmarks of aggressive disease and therapeutic resistance [22]. Clinically, GPAA1-high tumors correlated with shorter RFS, suggesting that GPAA1 may stratify patients at higher risk of recurrence who might benefit from intensified surveillance or adjuvant therapy. The absence of a significant OS association likely reflects confounding by heterogeneous post-recurrence treatments in public datasets.

At single-cell resolution, GPAA1 localized predominantly to malignant cells and was further elevated in peritoneal metastases relative to primary tumors. GPAA1-high malignant cells showed enrichment for oxidative phosphorylation, proteasome activity, and mitochondrial translation—consistent with a metabolically active state [23]. Metabolic reprogramming is increasingly recognized as a requirement for metastatic cells to meet the bioenergetic demands of dissemination and colonization [24,25]. The AKT pathway is a central regulator of survival, proliferation, and metastasis in CRC [20]. Our functional data demonstrate that GPAA1 modulates AKT phosphorylation, suggesting a potential signaling axis between GPI anchor biosynthesis and metastatic phenotypes. We propose that GPAA1-mediated GPI anchoring of specific cell surface receptors or adhesion molecules may enhance ligand–receptor interactions that converge on AKT activation, thereby promoting EMT and migration. However, because rescue experiments using AKT-specific inhibitors (e.g., MK-2206) or constitutively active AKT mutants were not performed, a direct causal relationship remains to be established. Identifying the specific GPI-anchored protein(s) that mediate this effect will be an important direction for future research.

CellChat analysis further revealed enhanced bidirectional crosstalk between GPAA1-high malignant cells and the microenvironment—outgoing signals to fibroblasts, B cells, T cells, and myeloid cells, and incoming signals from fibroblasts and endothelial cells. This reciprocal network suggests that GPAA1-high cells actively remodel the peritoneal niche, potentially promoting immune evasion, stromal activation, and vascular remodeling—all of which are critical for peritoneal implantation and outgrowth [26]. Spatial transcriptomics independently confirmed that GPAA1-positive malignant cells preferentially colocalize with stromal and myeloid compartments, reinforcing the notion of GPAA1 as a mediator of tumor–stromal interactions.

From a therapeutic perspective, GPAA1 itself may be targetable. A recent report demonstrated that GPAA1 inhibition abrogates the CD24 immune checkpoint in ovarian cancer [27], raising the possibility that GPAA1 blockade could have both direct anti-metastatic and immune-modulatory effects. However, the essential role of GPI anchoring in normal physiology mandates careful evaluation of on-target toxicity. Alternatively, the metabolic dependencies of GPAA1-high tumors—particularly oxidative phosphorylation—may render them susceptible to mitochondrial inhibitors such as biguanides [28]. Whether GPAA1 expression predicts response to stromal-targeting or immunotherapeutic agents also warrants investigation [29].

This research addresses a critical gap in the clinical identification of site-specific biomarkers for peritoneal metastasis. It offers a methodological advancement by integrating paired whole-exome sequencing, quantitative proteomics, single-cell transcriptomics, and spatial transcriptomics on matched primary and metastatic samples. This protein-centric approach serves as a complement to the predominantly transcriptomics-focused studies of colorectal cancer peritoneal metastasis.

This study is subject to several limitations. Firstly, the discovery cohort was relatively small, comprising 13 paired whole-exome sequencing samples and 7 paired proteomics samples. This limited sample size may have constrained the study’s ability to detect rare variants and diminished the generalizability of the findings. Additionally, the bulk WES and proteomic analyses are subject to potential tissue-composition confounding, as differences in the cellular composition between primary and metastatic tissues may contribute to the observed molecular differences. Moreover, the spatial transcriptomic analysis was conducted using a single tissue section, necessitating further validation of the observed GPAA1–stroma co-localization pattern in additional sections. Secondly, the precise mechanism by which GPAA1 activates AKT remains to be elucidated, and direct measurements of GPI-anchored substrates were not performed, so the GPI-anchored proteins downstream of GPAA1 remain to be identified. Third, the functional experiments were conducted exclusively in a single cell line (DLD1), and the animal studies were based on GPAA1 overexpression without in vivo loss-of-function validation. Additionally, the employment of immunodeficient (BALB/c nude) mice limits the assessment of T-cell-mediated immune evasion, leaving the proposed immunomodulatory effects of GPAA1 as hypothesis-generating and necessitating validation in immunocompetent models. Fourth, the differential expression analyses were performed at the single-cell level rather than the patient level, potentially exaggerating statistical significance due to within-sample correlation. Lastly, the survival analyses utilized publicly available cohorts with varied treatment modalities, underscoring the need for prospective validation in uniformly treated patient populations to ascertain the clinical utility of GPAA1 as a biomarker.

## 4. Materials and Methods

### 4.1. Patient Samples and Ethics

Paired primary colorectal cancer tissues and peritoneal metastatic tissues were collected from 13 patients who underwent surgical resection at The Second Affiliated Hospital, Zhejiang University. Matched adjacent normal tissues were obtained from a subset of patients. All samples were snap-frozen in liquid nitrogen immediately after resection and stored at −80 °C until further processing. Written informed consent was obtained from all patients, and the study was approved by the Institutional Review Board of The Second Affiliated Hospital, Zhejiang University School of Medicine (Ethics Approval No. 2022-0632; approved on 22 July 2022). The clinicopathological characteristics of the 23 patients are summarized in Table 2.

### 4.2. Whole-Exome Sequencing

Genomic DNA was extracted from frozen tissues using the QIAamp DNA Mini Kit (Qiagen, Hilden, Germany) according to the manufacturer’s protocol. Whole-exome sequencing was performed on 13 pairs of matched primary and peritoneal metastatic samples. Raw sequencing reads were aligned to the human reference genome (GRCh37/hg19) using BWA-MEM. Duplicate reads were marked using Picard, and base quality recalibration and variant calling were performed with GATK (v4.0). Somatic mutations were identified using MuTect2, and copy number alterations were analyzed using GATK CNV. Tumor mutational burden was calculated as the total number of non-synonymous somatic mutations per megabase. Mutation annotation and visualization were performed using Maftools (v2.8) in R (v4.0).

### 4.3. Quantitative Proteomics

Protein extraction and digestion were performed on 7 pairs of matched primary and peritoneal metastatic samples. Seven paired proteomic samples were selected from the 13 pairs based on tissue quality. Tissues were lysed in SDT buffer (4% SDS, 100 mM Tris-HCl, pH 7.6, 0.1 M DTT), and protein concentration was determined by the BCA assay. Proteins were digested with trypsin (Promega, Madison, WI, USA) at 37 °C overnight after reduction and alkylation. Differential protein expression analysis was performed using the limma package in R. Paired comparisons were conducted for the discovery cohort, and independent sample analysis for the validation cohort. Significantly differential proteins were defined as those with *p* < 0.05.

### 4.4. Single-Cell RNA Sequencing

Public single-cell RNA-seq data were obtained from two datasets: GSE183916 (5 peritoneal metastasis and 1 primary colorectal cancer samples) and GSE132465 (23 primary colorectal cancer and 10 normal mucosa samples). After quality control, a total of 23,389 cells were retained, comprising peritoneal metastasis samples (*n* = 5) and primary tumor samples (*n* = 5; 1 from GSE183916 and 4 from GSE132465), for downstream analysis, while normal mucosa samples were excluded. Data were integrated and batch-corrected. Downstream analysis was performed in R using Seurat (v4.0) [30]. Quality control filtering included removal of cells with <200 or >8000 detected genes, >15% mitochondrial reads, and <500 unique molecular identifiers. Data normalization was performed using the SCTransform method [31]. Dimensionality reduction, clustering, and UMAP visualization were conducted using standard Seurat workflows. Cell-type annotation was performed by mapping marker genes to established cell-type signatures, and malignant cells were identified based on the expression of colorectal cancer-specific epithelial marker genes (including EPCAM, KRT8, and KRT18). Differential gene expression analysis between GPAA1-high and GPAA1-low malignant cells was performed using the Wilcoxon rank-sum test. Cell–cell communication analysis was conducted using CellChat (v1.0) with default parameters [32].

### 4.5. Spatial Transcriptomics

Spatial transcriptomic data were retrieved from GEO (GSE225857) and analyzed using the 10x Visium platform workflow. Downstream analysis was performed in R using Seurat (v4.0). Cell-type deconvolution was conducted using RCTD [33]. Niche decomposition and spatial interaction analysis were performed using MISTy (v1.0) at three spatial scales: intra-niche (0-cell radius), juxta-5 (5-cell radius), and para-15 (15-cell radius).

### 4.6. Immunohistochemistry

IHC was performed on matched tissue sections from *n* = 10 patients. Formalin-fixed, paraffin-embedded tissue sections (3 um) were deparaffinized and rehydrated. Antigen retrieval was performed in citrate buffer (pH 6.0) at 95 °C for 20 min. Endogenous peroxidase activity was blocked with 3% hydrogen peroxide for 10 min. Sections were incubated with primary antibodies against GPAA1 (1:200, Proteintech, Wuhan, China, 10104-1-AP), MUC16 (1:200, Proteintech, Wuhan, China, 20077-1-AP), and FAT4 (1:200, Sangon Biotech, Shanghai, China, D261201) at 4 °C overnight. Signal detection was performed using the DAB kit (Aladdin, Shanghai, China, R43443) according to the manufacturer’s protocol. Sections were counterstained with hematoxylin, dehydrated, and mounted. Images were acquired using a Nikon Eclipse Ni-E microscope. Immunohistochemical staining was scored blinded to clinical information. Stained sections were scanned and analyzed using ImageJ. For each section, five hotspots with the highest staining were selected and scored, and the final score was taken as the mean of the five hotspot scores. The H-score was calculated as (% area of weak staining) + (2 × % area of moderate staining) + (3 × % area of strong staining), yielding a range of 0–300 [34].

### 4.7. Public Data Analysis and Survival Analysis

GPAA1 mRNA expression data and clinical information for colorectal cancer were retrieved from The Cancer Genome Atlas (TCGA) and Gene Expression Omnibus (GEO) databases. Public single-cell transcriptomic datasets (GSE132465, GSE183916) and spatial transcriptomic data (GSE225857) were integrated for downstream analyses. Patients were stratified into GPAA1-high and GPAA1-low groups based on median expression. Differential gene expression analysis was performed using DESeq2 [35]. KEGG and Hallmark pathway enrichment analyses were conducted using clusterProfiler (v3.18) [36]. Univariable Cox proportional hazards regression (log-rank) was performed via the Kmplot platform (https://kmplot.com/analysis/, 17 March 2026) to calculate hazard ratios and 95% confidence intervals for RFS and OS. In addition, univariable and multivariable Cox proportional hazards regression was performed using TCGA-COAD clinical data to assess the association of GPAA1 with disease-specific survival (DSS); GPAA1 expression group (high vs. low), stage, age, and sex were included as covariates in the multivariable model.

### 4.8. Cell Culture and Reagents

Human colorectal cancer cell lines (DLD1, RKO, LOVO, SW620, SW480, HT29, HCT116) and the normal colonic epithelial cell line NCM460 were cultured in RPMI-1640 or DMEM (Gibco, Grand Island, NY, USA) supplemented with 10% fetal bovine serum (Gibco, Grand Island, NY, USA) and 1% penicillin–streptomycin at 37 °C in a humidified atmosphere with 5% CO_2_.

### 4.9. Plasmid Construction and Lentiviral Transduction

The full-length GPAA1 coding sequence was cloned into the PLV3 lentiviral expression vector to generate PLV3-GPAA1. Two independent shRNA sequences targeting GPAA1 (ShGPAA1-1: GAGGTCTACACGCAGAGTTTC; ShGPAA1-2: TGAGACCCACGAGCGCTATAT) and a non-targeting scramble control (ShNT) were cloned into the pLKO.1-puro vector. Lentiviral particles were produced by co-transfecting 293T cells with the target plasmid, psPAX2 packaging plasmid, and pMD2.G envelope plasmid using Lipofectamine 3000 (Thermo Fisher, Waltham, MA, USA). Viral supernatants were collected at 48 and 72 h, filtered through 0.45 um membranes, and used to infect DLD1 cells in the presence of 8 ug/mL polybrene. Stable cell lines were selected with 2 ug/mL puromycin for 7 days.

### 4.10. Western Blot Analysis

Cells or tissues were lysed in RIPA buffer (Yeasen Biotechnology, Shanghai, China) supplemented with protease and phosphatase inhibitors. Protein concentrations were determined by the BCA assay. Equal amounts of protein (20 ug) were separated by 8–12% SDS-PAGE and transferred to PVDF membranes (Millipore, Burlington, MA, USA). Membranes were blocked with 5% non-fat milk in TBST for 1 h at room temperature and incubated with primary antibodies overnight at 4 °C. Primary antibodies included: GPAA1 (1:1000, Proteintech, Wuhan, China, 10104-1-AP), E-cadherin (1:1000, Zenbio, Chengdu, China, 340341), N-cadherin (1:1000, Zenbio, Chengdu, China, 680441), Vimentin (1:1000, Zenbio, Chengdu, China, 22775), AKT (1:1000, Huabio, Hangzhou, China, HA721870), p-AKT (Ser473, 1:1000, Huabio, HA721115), and beta-Actin (1:5000, Proteintech, Wuhan, China, 66009-1-Ig). HRP-conjugated secondary antibodies (1:5000, Huabio, Hangzhou, China) were applied for 1 h at room temperature. Signals were detected using the ECL chemiluminescence kit (Thermo Fisher, Waltham, MA, USA) and visualized on a ChemiDoc XRS+ imaging system (Bio-Rad, Hercules, CA, USA).

### 4.11. Quantitative RT-PCR

Total RNA was extracted using TRIzol reagent (Thermo Fisher, Waltham, MA, USA) according to the manufacturer’s protocol. cDNA was synthesized using the PrimeScript RT Reagent Kit (Takara, Kusatsu, Shiga, Japan). Quantitative PCR was performed on the QuantStudio 5 Real-Time PCR System (Applied Biosystems) using SYBR Green Premix Ex Taq (Takara, Kusatsu, Shiga, Japan). GPAA1 primers: forward AGCAGTGATGTGGTCACCAG, reverse CGGTCCATCCAATGATGTCCA. GAPDH primers: forward ACAACTTTGGTATCGTGGAAGG, reverse GCCATCACGCCACAGTTTC. Relative GPAA1 mRNA expression was calculated using the 2^−ΔΔCt^ method with GAPDH as the endogenous reference. The vector control (for GPAA1 overexpression) and the non-targeting control (ShNT, for knockdown) served as the calibrator groups, which were set to a relative expression of 1.

### 4.12. Cell Proliferation Assay

Cell proliferation was assessed using the Cell Counting Kit-8 (CCK-8, Yeasen Biotechnology, Shanghai, China). Cells were seeded in 96-well plates at 2000 cells per well and cultured for 0, 1, 2, 3, and 4 days. At each time point, 10 uL CCK-8 solution was added to each well and incubated for 2 h at 37 °C. Absorbance at 450 nm was measured using a microplate reader (BioTek Synergy H1). Experiments were performed in triplicate and repeated three times independently.

### 4.13. Colony-Formation Assay

Cells were seeded in 6-well plates at 500 cells per well and cultured for 14 days. Colonies were fixed with 4% paraformaldehyde for 15 min and stained with 0.1% crystal violet for 30 min. Excess dye was washed away with PBS, and colonies were imaged and counted using ImageJ (version 1.54g) software.

### 4.14. Wound-Healing Assay

Cells were seeded in 6-well plates and cultured to confluence. A straight scratch was made across the monolayer using a 200 uL pipette tip. Detached cells were washed away with PBS, and cultures were maintained in serum-free medium. Wound closure was imaged at 0 and 24 h using an inverted microscope (Olympus IX73). The relative wound area was quantified using ImageJ.

### 4.15. Transwell Migration Assay

Transwell inserts (8 um pore size, Corning, Corning, NY, USA) were placed in 24-well plates. Cells (2 × 10^4^) in 200 uL serum-free medium were seeded in the upper chamber, and 600 uL medium containing 10% FBS was added to the lower chamber as a chemoattractant. After 24 h, non-migrated cells on the upper surface were removed with a cotton swab. Migrated cells on the lower surface were fixed with 4% paraformaldehyde, stained with 0.1% crystal violet, and imaged under a microscope.

### 4.16. Tumor Xenograft Experiments

Xenograft models were established using female BALB/c-Nude mice (purchased from Jiangsu Jicui Yaokang Biotechnology Co., Ltd. (Nanjing, Jiangsu, China).; body weight 20 g, 4–6 weeks old), randomly assigned to groups (*n* = 5 per group). For the subcutaneous model, DLD1-Vector or DLD1-GPAA1-OE cells (1 × 10^7^ cells in 100 μL PBS:Matrigel = 1:1) were injected subcutaneously into the right flank. For the intraperitoneal metastasis model, cells (1 × 10^7^ cells in 200 μL PBS) were injected intraperitoneally. Tumor length and width were measured every 2 days from day 10 post-inoculation using a vernier caliper. Tumor volume was calculated as V = (W^2^ × L)/2. The subcutaneous experiment was terminated on day 20 to ensure tumor diameter remained below the ethical limit of 15 mm. For the intraperitoneal model, samples were collected on day 21. Group allocation was conducted using a random-number table, ensuring that the investigator responsible for daily animal care and tumor/nodule measurement remained blinded to the group assignments. A sample size of *n* = 5 was employed, aligning with standard practices for subcutaneous and peritoneal xenograft models. Inclusion criteria specified healthy 20-g, 4–6-week-old female mice that had successfully completed model construction, while exclusion criteria included accidental death, failure to establish tumors, or severe infection. In the subcutaneous model, endpoint tumors were excised and weighed. In the intraperitoneal metastasis model, visible peritoneal nodules were harvested and weighed at the endpoint to quantify the total tumor burden. Nodules from each mouse were individually harvested and weighed. All animal experimental protocols were approved by the Animal Ethics Committee of the Second Affiliated Hospital, Zhejiang University School of Medicine (Approval No. 2026-187, approved on 30 June 2026), and were conducted in accordance with the ARRIVE guidelines 2.0 and the National Institutes of Health Guide for the Care and Use of Laboratory Animals.

### 4.17. Statistical Analysis

All statistical analyses were performed in R (v4.0) or GraphPad Prism (v9.0). Data are presented as mean +/− standard deviation (SD) unless otherwise specified. Comparisons between two groups were performed using the paired or unpaired Student’s *t*-test or Wilcoxon rank-sum test as appropriate. Comparisons among multiple groups were performed using one-way ANOVA followed by Tukey’s post hoc test. Survival curves were compared using the log-rank test. For all in vitro assays, results are reported as the number of independent experiments (*n* = 3); technical replicates within an independent experiment were averaged and were not treated as independent observations. Normality and homogeneity of variance were assessed using the Shapiro–Wilk test and Levene test, respectively; when these assumptions were violated, the non-parametric Wilcoxon rank-sum test (two groups) or Kruskal–Wallis test (multiple groups) was used instead. For differential proteomics, differentially expressed proteins were defined at a nominal *p* < 0.05 threshold. For single-cell RNA-seq, differential expression testing used the Wilcoxon rank-sum test with Bonferroni correction for display purposes. *p* < 0.05 was considered statistically significant. * *p* < 0.05, ** *p* < 0.01, *** *p* < 0.001, **** *p* < 0.0001.

## 5. Conclusions

In summary, using an integrated multi-omic approach, we identified GPAA1 as an upregulated protein in CRC peritoneal metastasis. High GPAA1 expression correlates with shorter RFS and metabolic pathway activation. Single-cell and spatial transcriptomics demonstrate that GPAA1 marks a metabolically active malignant cell state with enhanced tumor–stromal crosstalk. Functional studies confirm that GPAA1 promotes proliferation, migration, and EMT in association with AKT phosphorylation, establishing it as a candidate marker with growth-promoting effects in CRC peritoneal metastasis.

## Figures and Tables

**Figure 1:**
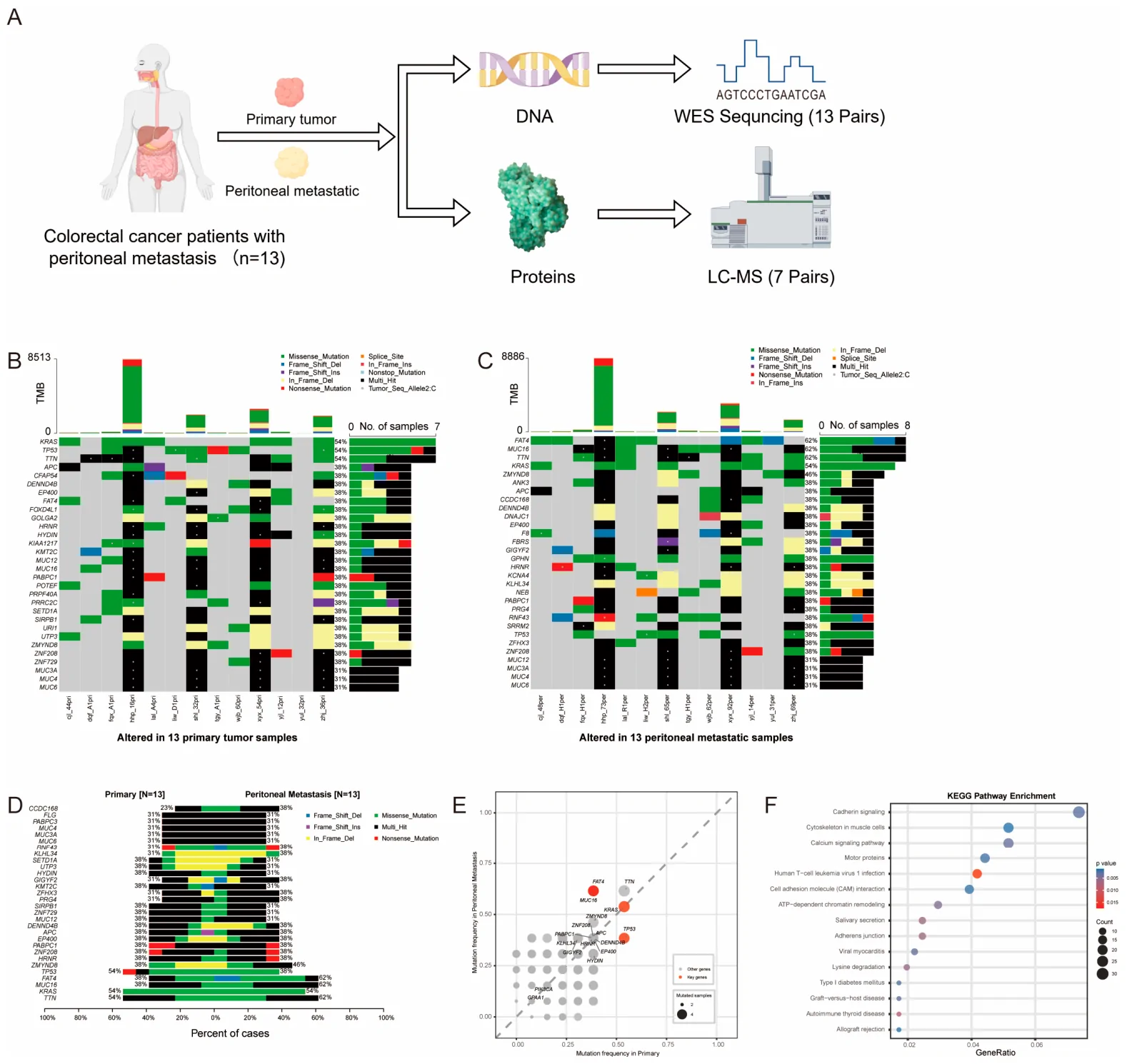
Multi-omic profiling of paired primary and peritoneal metastatic colorectal cancer samples. (**A**) Schematic workflow of the study. Whole-exome sequencing (WES) was performed on 13 pairs of matched primary tumors and peritoneal metastases from colorectal cancer patients. Label-free quantitative proteomics by LC-MS was conducted on 7 pairs of these samples. (**B**,**C**) OncoPrint plots showing the mutational landscape of the top altered genes in (**B**) 13 primary tumor samples and (**C**) 13 matched peritoneal metastatic samples. Mutation types are color-coded as indicated. TMB—tumor mutational burden. (**D**) Comparative bar plot displaying the mutation frequency of selected genes in primary versus peritoneal metastatic tumors. Genes are ranked by differential mutation prevalence between the two groups. (**E**) Scatter plot comparing mutation frequencies in primary (*x*-axis) and peritoneal metastatic (*y*-axis) tumors. Genes with significantly higher mutation frequency in metastases are highlighted in red. Dot size represents the number of mutated samples. (**F**) KEGG pathway enrichment analysis of differentially expressed proteins between primary and peritoneal metastatic lesions. The *x*-axis indicates gene ratio; dot size and color represent gene count and adjusted *p*-value, respectively.

**Figure 2:**
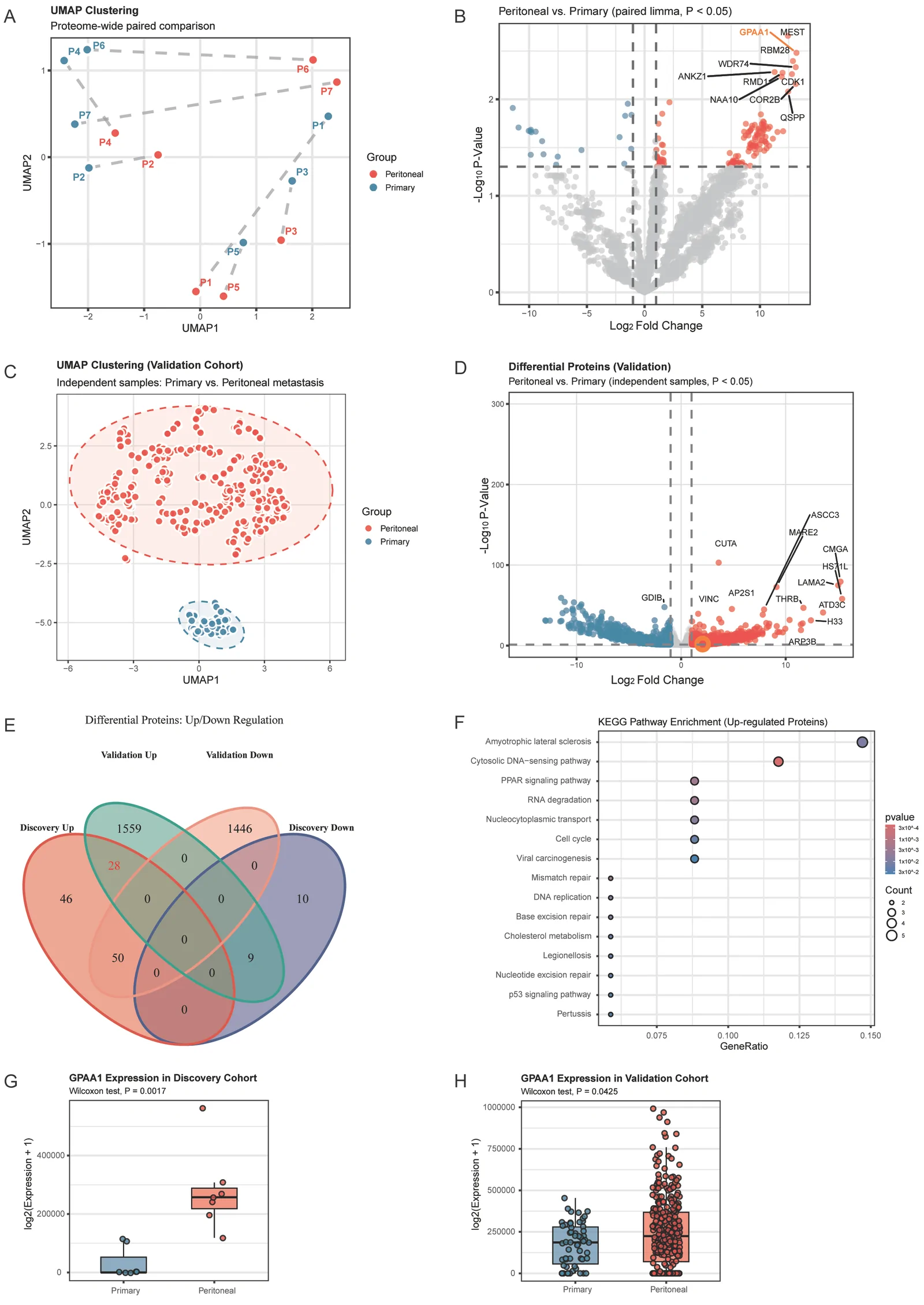
Proteomic discovery and validation of GPAA1 as an up-regulated protein in colorectal cancer peritoneal metastasis. (**A**) UMAP clustering of proteome-wide paired comparison between matched primary (blue) and peritoneal metastatic (red) samples from the discovery cohort (*n* = 7 pairs). Dashed lines connect paired samples from individual patients (P1–P7). (**B**) Volcano plot of differentially expressed proteins between peritoneal metastasis and primary tumors (paired limma, *p* < 0.05, *n* = 7 pairs). Significantly up-regulated proteins (log2 fold change > 0, −log10 *p*-value > threshold) are highlighted in red; down-regulated proteins in blue. Key up-regulated proteins including GPAA1 are labeled. (**C**) UMAP clustering of an independent validation cohort (*n* = 334 primary and 58 peritoneal metastatic samples) comparing primary (blue) and peritoneal metastatic (red) samples. Dashed ellipses indicate group distributions. (**D**) Volcano plot of differential proteins in the validation cohort (independent samples, *n* = 334 primary and 58 peritoneal metastatic, *p* < 0.05). Significantly up-regulated proteins are highlighted in red; down-regulated proteins in blue. (**E**) Venn diagram showing the overlap of up-regulated and down-regulated proteins between discovery and validation cohorts. Numbers indicate protein counts in each category. (**F**) KEGG pathway enrichment analysis of up-regulated proteins in peritoneal metastases. The *x*-axis indicates gene ratio; dot size and color represent gene count and adjusted *p*-value, respectively. (**G**,**H**) Box plots comparing GPAA1 protein expression levels between primary and peritoneal metastatic samples in (**G**) the discovery cohort (*n* = 7 pairs; Wilcoxon test, *p* = 0.0017) and (**H**) the validation cohort (*n* = 334 primary and 58 peritoneal metastatic; Wilcoxon test, *p* = 0.0425). Individual data points are overlaid.

**Figure 3:**
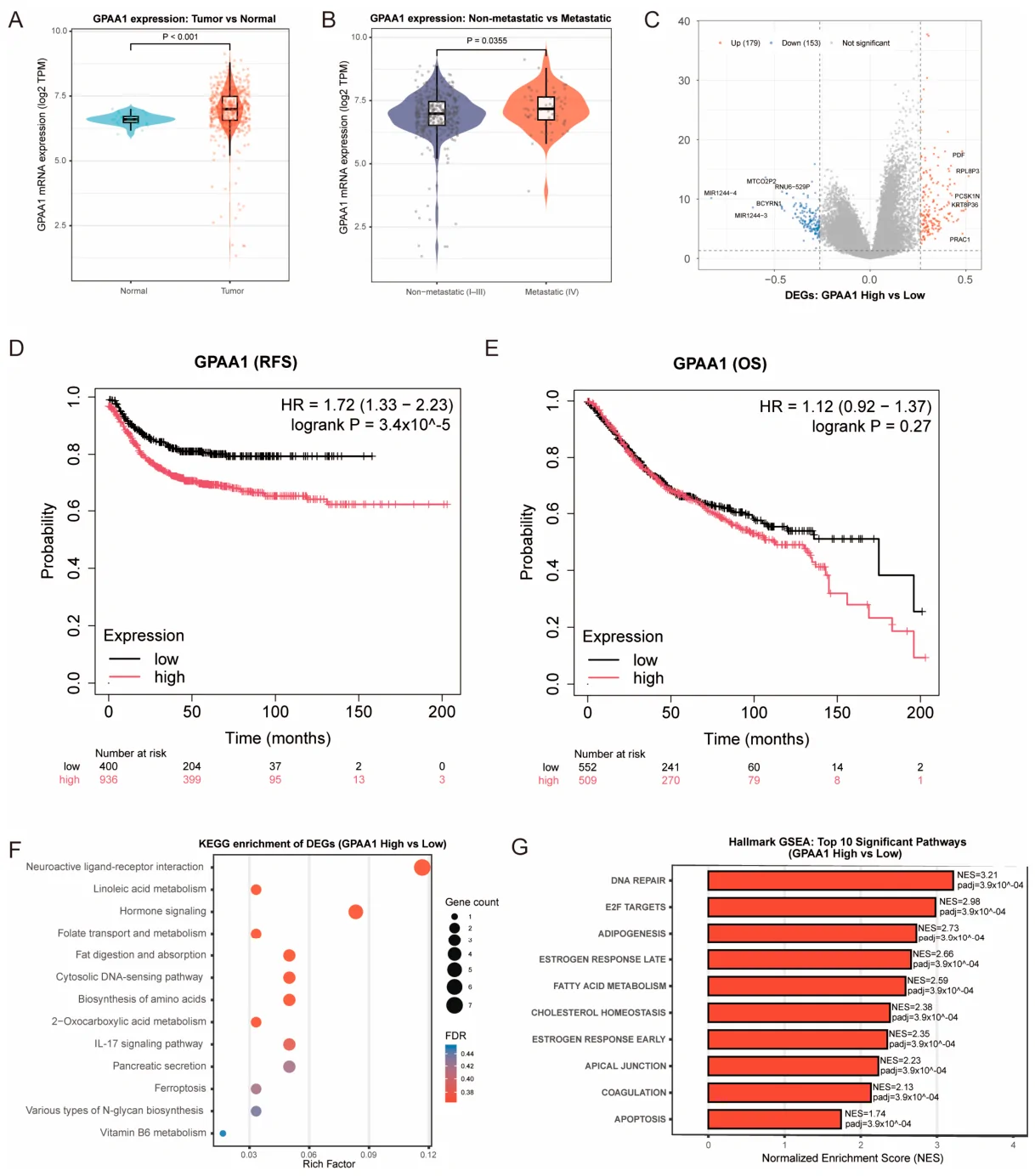
Clinical significance and pathway enrichment of GPAA1 in colorectal cancer. (**A**) Violin plot comparing GPAA1 mRNA expression between colorectal cancer tumor (*n* = 473) and adjacent normal (*n* = 41) tissues (Wilcoxon test, *p* < 0.001). (**B**) Violin plot comparing GPAA1 mRNA expression between non-metastatic (stage I–III, *n* = 383) and metastatic (stage IV, *n* = 65) colorectal cancer tumors (Wilcoxon test, *p* = 0.0355). (**C**) Volcano plot of differentially expressed genes (DEGs) between GPAA1-high and GPAA1-low expression groups. Up-regulated genes (179) are shown in red; down-regulated genes (153) in blue; non-significant genes in gray. (**D**,**E**) Kaplan–Meier survival curves for (**D**) recurrence-free survival (RFS) and (**E**) overall survival (OS) stratified by GPAA1 expression level. High GPAA1 expression is associated with significantly shorter RFS (HR = 1.72, 95% CI: 1.33–2.23, log-rank *p* = 3.4 × 10^−5^) but not OS (HR = 1.12, 95% CI: 0.92–1.37, log-rank *p* = 0.27). The KM-plot was obtained from the Kmplot website. (**F**) KEGG pathway enrichment analysis of DEGs between GPAA1-high and GPAA1-low groups. The *x*-axis indicates rich factor; dot size and color represent gene count and false discovery rate (FDR), respectively. (**G**) Hallmark GSEA of the top 10 significant pathways enriched in GPAA1-high tumors. NES—normalized enrichment score; padj—adjusted *p*-value.

**Figure 4:**
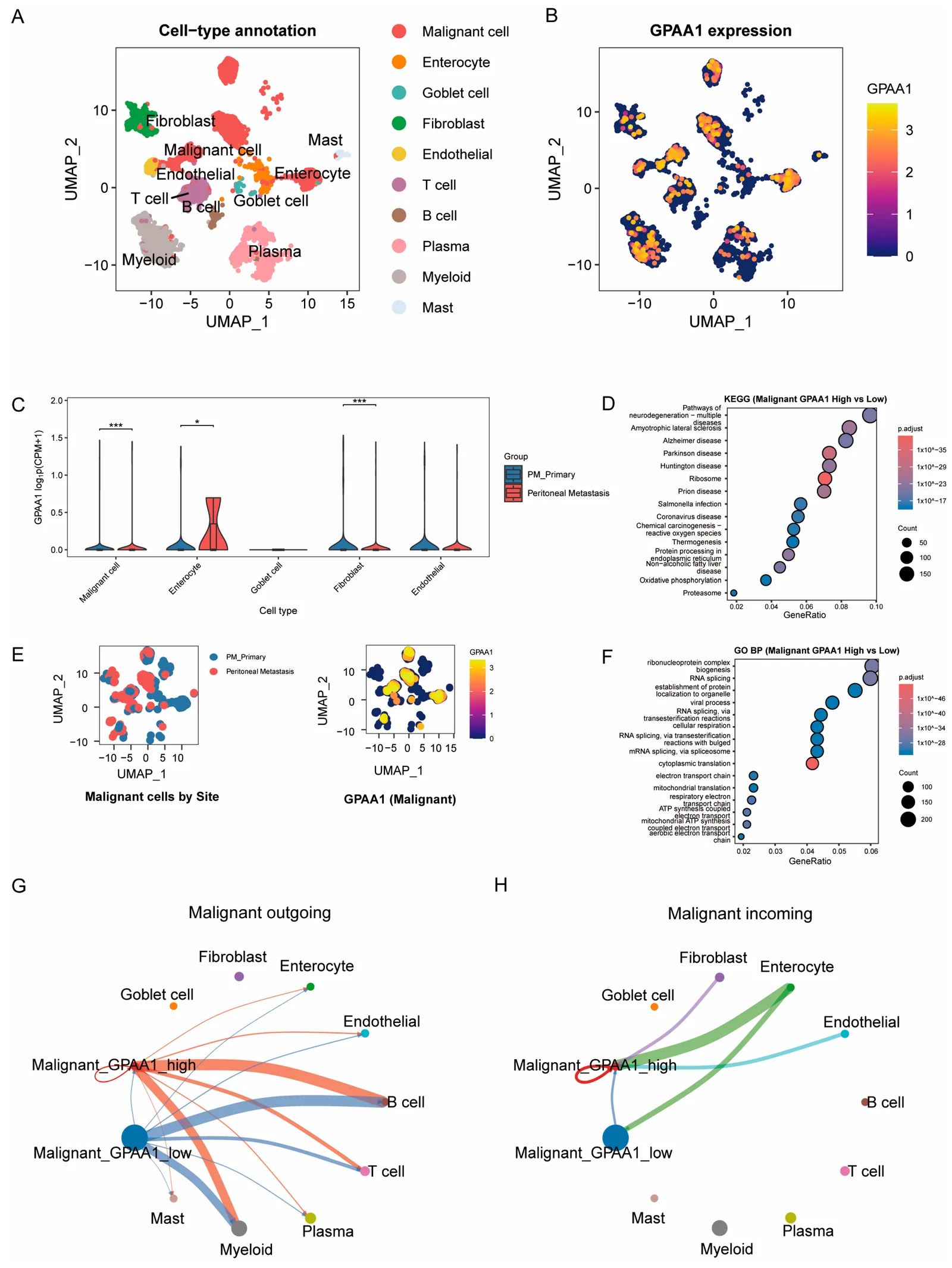
Single-cell transcriptomic analysis of GPAA1 in the colorectal cancer peritoneal metastatic microenvironment. (**A**) UMAP plot of single-cell RNA sequencing data showing cell-type annotation. Ten major cell types are color-coded: malignant cell, enterocyte, goblet cell, fibroblast, endothelial, T cell, B cell, plasma, myeloid, and mast. (**B**) Feature plot showing GPAA1 expression levels across all cell clusters. Color intensity represents GPAA1 expression level. (**C**) Violin plot comparing GPAA1 expression across cell types between primary tumors (PM_Primary, blue) and peritoneal metastases (Peritoneal Metastasis, red). (**D**) KEGG pathway enrichment analysis of differentially expressed genes in malignant cells stratified by GPAA1 expression (GPAA1-high vs. GPAA1-low). The *x*-axis indicates gene ratio; dot size and color represent gene count and adjusted *p*-value, respectively. (**E**) UMAP plots of malignant cells. **Left**: cells colored by tissue origin (PM_Primary in blue, Peritoneal Metastasis in red). **Right**: GPAA1 expression levels in malignant cells. (**F**) Gene Ontology (GO) biological process enrichment analysis of differentially expressed genes in GPAA1-high versus GPAA1-low malignant cells. The *x*-axis indicates gene ratio; dot size and color represent gene count and adjusted *p*-value, respectively. (**G**,**H**) CellChat analysis of cell–cell communication networks. (**G**) Outgoing signaling from malignant cells (GPAA1-high and GPAA1-low) to other cell types in the tumor microenvironment. (**H**) Incoming signaling to malignant cells (GPAA1-high and GPAA1-low) from other cell types. Line thickness represents interaction strength. * *p* < 0.05, *** *p* < 0.001.

**Figure 5:**
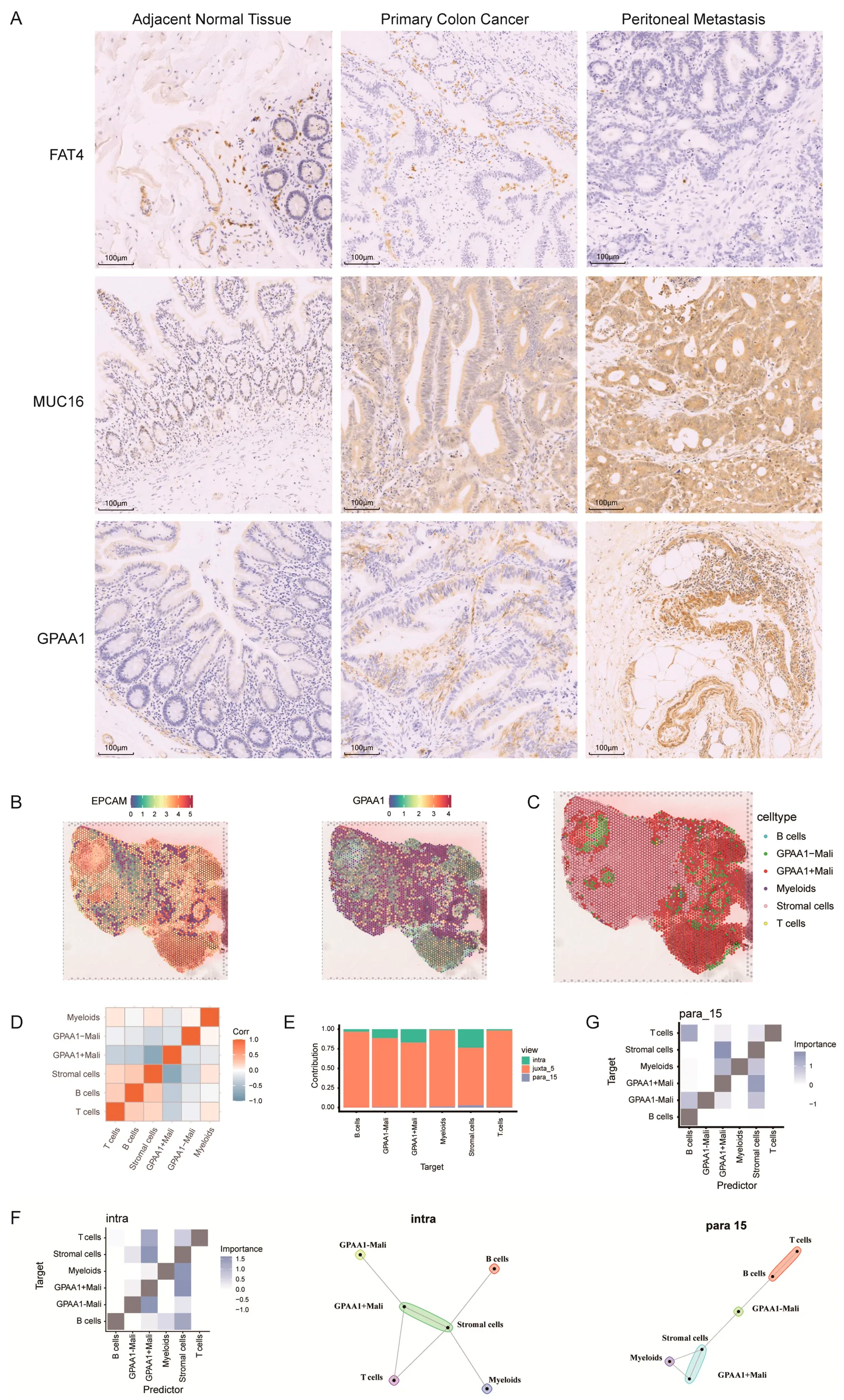
Immunohistochemical validation and spatial transcriptomic analysis of GPAA1 in colorectal cancer peritoneal metastasis. (**A**) Representative immunohistochemical staining images of FAT4, MUC16, and GPAA1 in matched adjacent normal tissue, primary colon cancer, and peritoneal metastasis. Representative images are shown from *n* = 10 independent samples. Scale bar, 100 μm. (**B**) Spatial transcriptomic feature plots showing EPCAM expression (**left**) and GPAA1 expression (**middle**) across a peritoneal metastatic tissue section. Color intensity represents expression level. (**C**) Spatial cell-type annotation map identifying distinct cell populations: B cells, GPAA1-negative malignant cells (GPAA1-Mali), GPAA1-positive malignant cells (GPAA1+Mali), myeloid cells, stromal cells, and T cells. B and C are derived from the same 10x Visium tissue section. (**D**) Correlation heatmap of spatial co-localization between different cell types. Color intensity represents Pearson correlation coefficient (Cor). (**E**) MISTy niche decomposition analysis showing the relative contribution of intra-niche (intra), juxta-5 (juxta_5, 5-cell radius), and para-15 (para_15, 15-cell radius) signaling across different cell-type niches. (**F**) Intra-niche interaction importance heatmap (**left**) and network visualization (**middle**) showing cell–cell communication within the intra-niche spatial scale. (**G**) Para_15 interaction importance heatmap (**top**) and network visualization (**bottom**) showing cell–cell communication at the 15-cell radius spatial scale.

**Figure 6:**
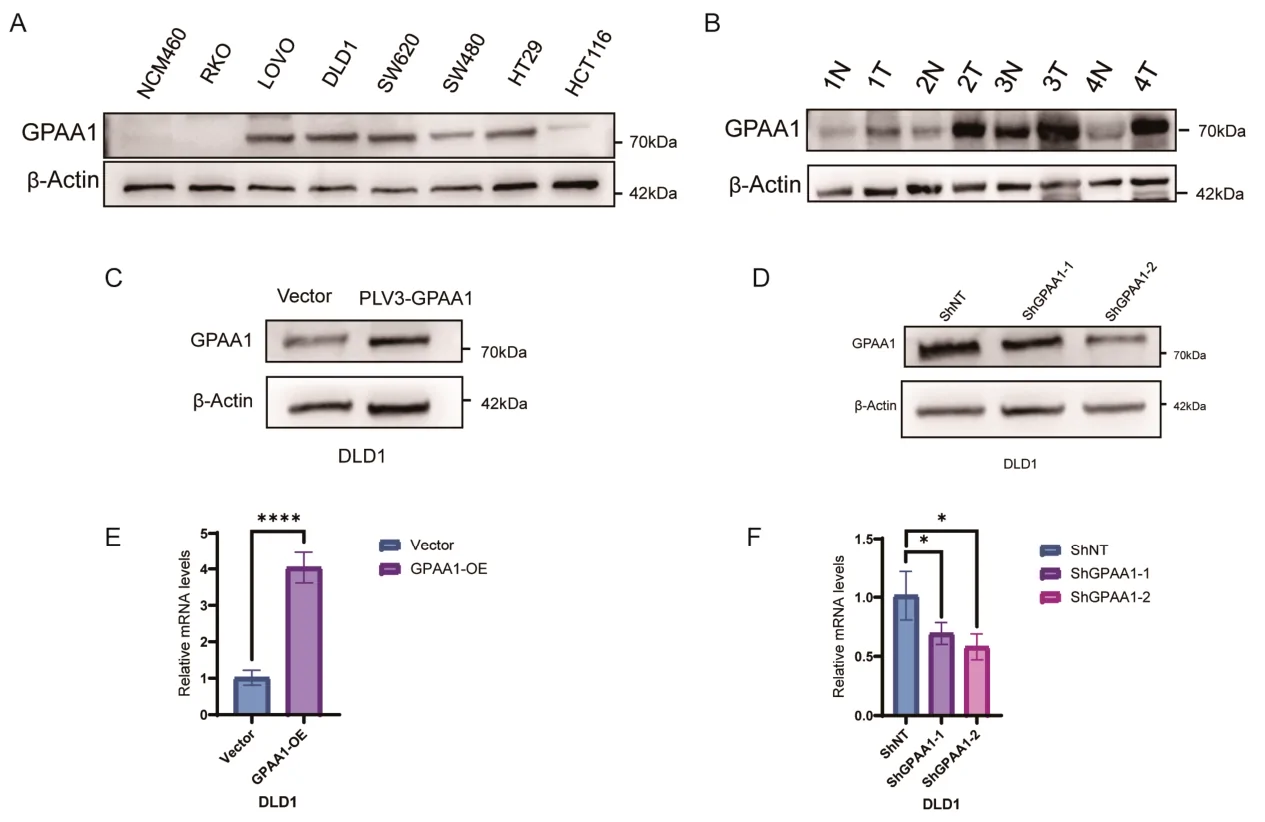
Endogenous GPAA1 expression in colorectal cancer cell lines and patient tissues, and generation of GPAA1-modulated cell models. (**A**) Western blot analysis of endogenous GPAA1 protein expression in a panel of colorectal cancer cell lines (RKO, LOVO, DLD1, SW620, SW480, HT29, HCT116) and the normal colonic epithelial cell line NCM460. β-Actin served as loading control. GPAA1 molecular weight: ~70 kDa. (**B**) Western blot analysis of GPAA1 protein expression in matched tumor (T) and adjacent normal (N) tissues from four colorectal cancer patients (1–4). β-Actin served as loading control. (**C**) Western blot validation of GPAA1 overexpression in DLD1 cells transduced with PLV3-GPAA1 lentiviral vector compared to vector control. β-Actin served as loading control. (**D**) Western blot validation of GPAA1 knockdown in DLD1 cells transduced with two independent shRNA sequences (ShGPAA1-1 and ShGPAA1-2) compared to ShNT non-targeting control. β-Actin served as loading control. (**E**) Quantitative RT-PCR analysis of GPAA1 mRNA levels in vector control and GPAA1-overexpressing (GPAA1-OE) DLD1 cells. Data are presented as mean ± SD from three independent experiments (*n* = 3). Statistical analyses were determined by Student’s *t*-test. (**F**) Quantitative RT-PCR analysis of GPAA1 mRNA levels in ShNT control, ShGPAA1-1, and ShGPAA1-2 DLD1 cells. Data are presented as mean ± SD from three independent experiments (*n* = 3). Statistical analyses were determined by Student’s *t*-test. * *p* < 0.05, **** *p* < 0.0001.

**Figure 7:**
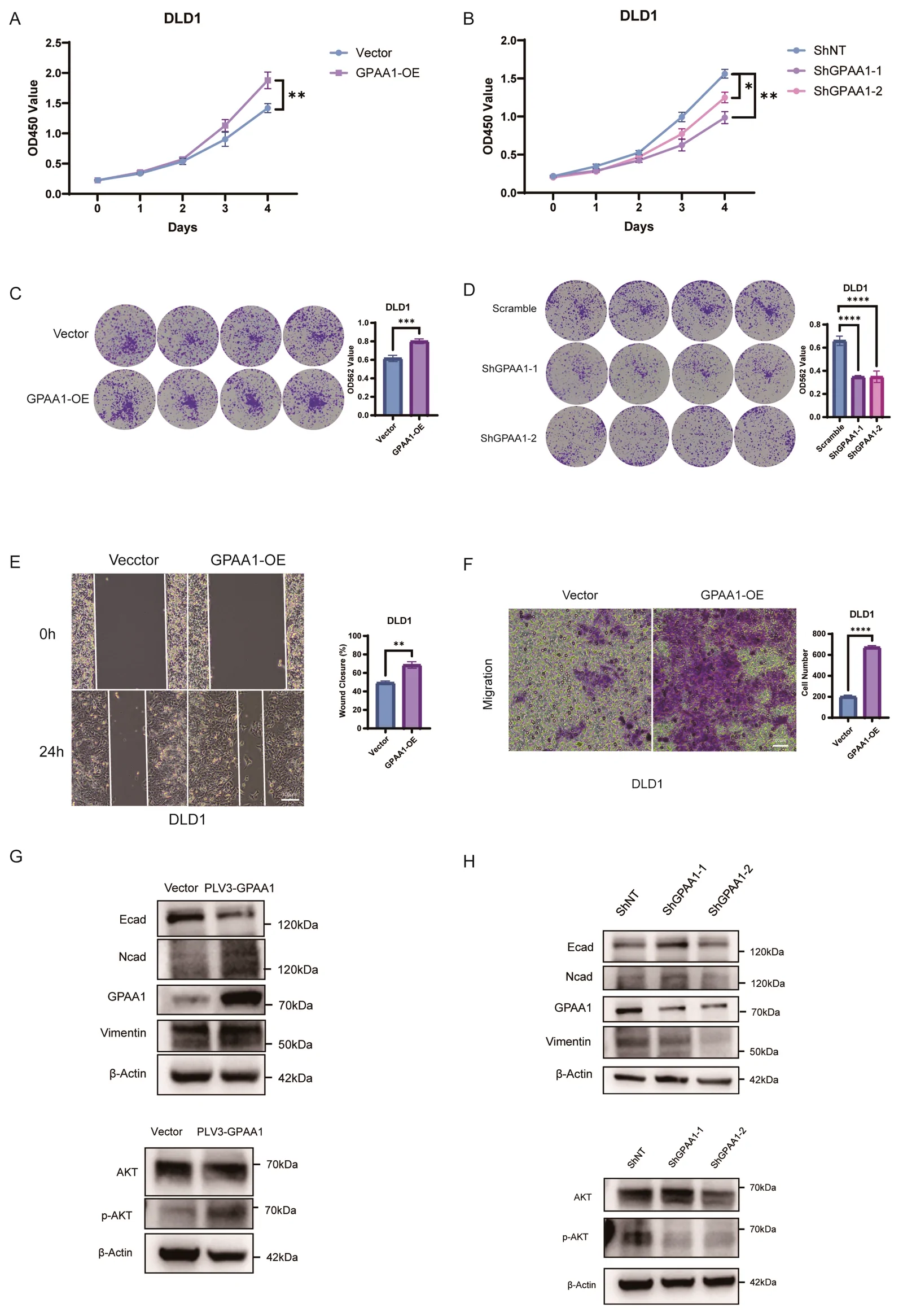
GPAA1 promotes colorectal cancer cell proliferation, migration, and EMT in association with AKT phosphorylation. (**A**,**B**) CCK-8 proliferation assay of (**A**) vector control and GPAA1-overexpressing (GPAA1-OE) DLD1 cells, and (**B**) ShNT control, ShGPAA1-1, and ShGPAA1-2 DLD1 cells over 4 days. Data are presented as mean ± SD from three independent experiments (*n* = 3). Statistical analyses were determined by two-way ANOVA followed by Tukey’s multiple comparison test. (**C**,**D**) Representative images of colony-formation assays (*n* = 3 independent experiments) for (**C**) vector and GPAA1-OE cells, and (**D**) Scramble, ShGPAA1-1, and ShGPAA1-2 cells. Statistical analyses were determined by one-way ANOVA followed by Tukey’s post hoc test. (**E**) Wound-healing assay images at 0 h and 24 h showing the migratory capacity of control (Ctrl) and GPAA1-OE DLD1 cells. Three independent experiments were performed (*n* = 3). Scale bar indicated. Statistical analyses were determined by one-way ANOVA followed by Tukey’s post hoc test. (**F**) Transwell migration assay images comparing migrated cell numbers between Ctrl and GPAA1-OE DLD1 cells (*n* = 3 independent experiments). Statistical analyses were determined by one-way ANOVA followed by Tukey’s post hoc test. (**G**) Western blot analysis of EMT markers (E-cadherin, N-cadherin, Vimentin), GPAA1, and AKT signaling components (AKT, p-AKT) in vector control and PLV3-GPAA1 DLD1 cells. (**H**) Western blot analysis of EMT markers and AKT signaling components in ShNT, ShGPAA1-1, and ShGPAA1-2 DLD1 cells. β-Actin served as loading control. * *p* < 0.05, ** *p* < 0.01, *** *p* < 0.001, **** *p* < 0.0001.

**Figure 8:**
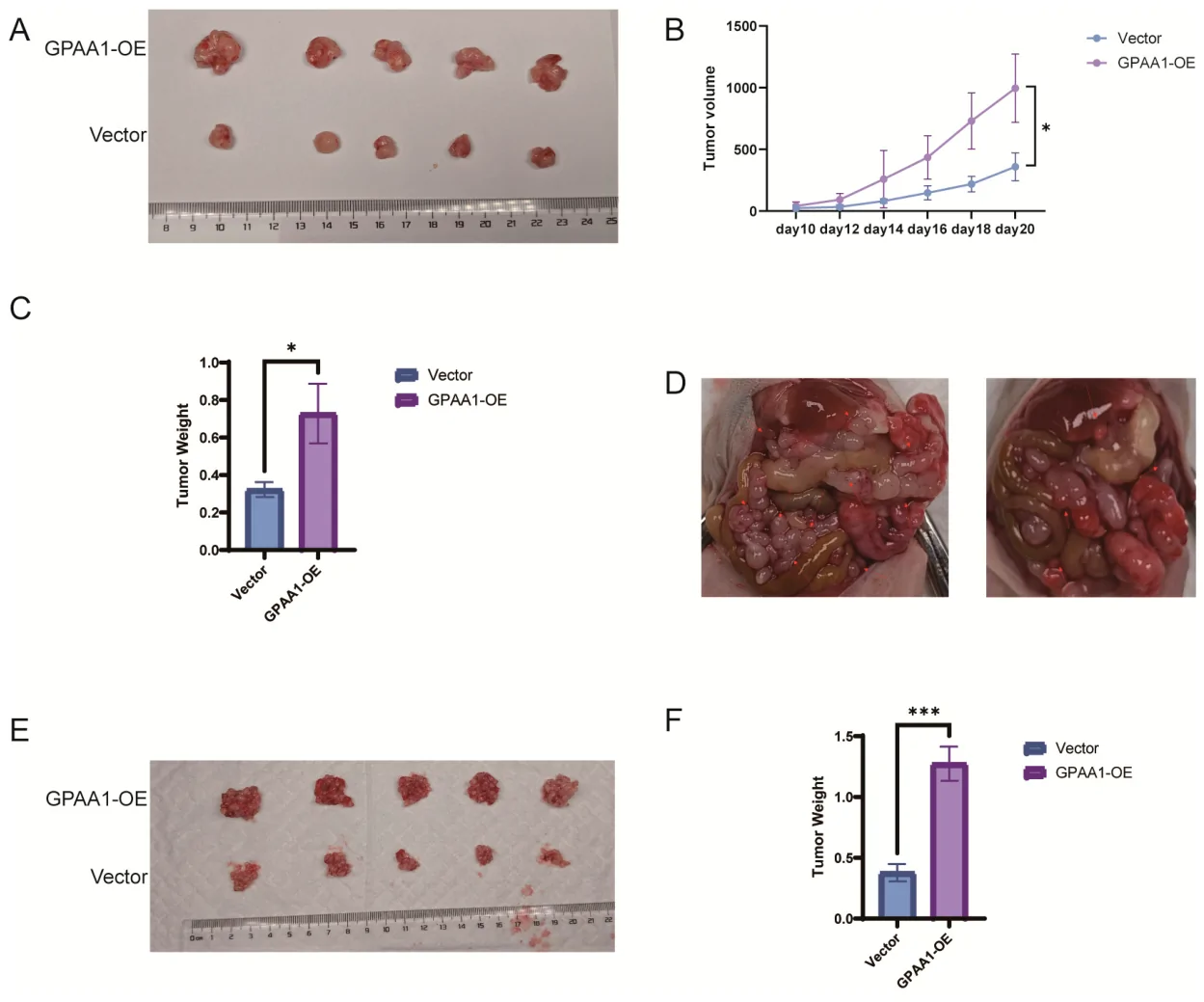
GPAA1 overexpression promotes subcutaneous tumor growth and peritoneal metastasis of colorectal cancer. (**A**) DLD1-Vector and DLD1-GPAA1-OE cells were subcutaneously inoculated into the right flank of BALB/c nude mice (*n* = 5 per group). Mice were sacrificed and tumors were excised on day 20. (**B**) Subcutaneous tumor growth curves. Tumor length and width were measured every 2 days starting from day 10 post-inoculation, and tumor volume was calculated using the formula V = (L × W^2^)/2. Tumor volumes in the GPAA1-OE group were significantly larger than those in the Vector group. (**C**) Endpoint subcutaneous tumor weights. Tumor weights in the GPAA1-OE group were significantly higher than those in the Vector group. (**D**) Representative gross anatomical images of the peritoneal metastasis model. DLD1-Vector and DLD1-GPAA1-OE cells were inoculated via intraperitoneal injection. Diffuse metastatic nodules were observed throughout the peritoneal cavity in the GPAA1-OE group (indicated by arrows), whereas fewer metastatic foci were observed in the Vector group. (**E**) All metastatic nodules isolated from the peritoneal cavities of mice in both groups. Both the number and size of nodules in the GPAA1-OE group were markedly greater than those in the Vector group. (**F**) Total peritoneal metastatic tumor weights. The total weight of peritoneal metastatic tumors in the GPAA1-OE group was significantly higher than that in the Vector group. Data are presented as mean ± SD. Statistical analyses were determined by one-way ANOVA followed by Tukey’s post hoc test. * *p* < 0.05, *** *p* < 0.001.

**Table 1:** Clinicopathological characteristics of the proteomic validation cohort.

Characteristic	Validation Cohort (*n* = 392)
Age (years), median (range)	61 (19–91)
Sex, *n* (%)	
Male	231 (58.9%)
Female	161 (41.1%)
Primary tumor site, *n* (%)	
Right-sided	198 (50.5%)
Left-sided	164 (41.8%)
Transverse colon	30 (7.7%)
Histological grade, *n* (%)	
Well differentiated	88 (22.4%)
Moderately differentiated	198 (50.5%)
Poorly differentiated	56 (14.3%)
Mucinous	31 (7.9%)
Signet-ring cell	12 (3.1%)
Unspecified/other	7 (1.8%)

**Table 2:** Clinicopathological characteristics of the study cohort.

Characteristic	All Patients (*n* = 23)	WES + Proteomics (*n* = 7)	WES Only (*n* = 6)	IHC (*n* = 10)
Age (years), median (range)	53 (28–88)	38 (35–88)	56 (28–72)	55.5 (31–81)
**Sex, *n* (%)**				
Male	7 (30.4%)	0 (0%)	4 (66.7%)	3 (30.0%)
Female	16 (69.6%)	7 (100%)	2 (33.3%)	7 (70.0%)
**Primary tumor site, *n* (%)**				
Left-sided	11 (47.8%)	2 (28.6%)	5 (83.3%)	4 (40.0%)
Right-sided	6 (26.1%)	3 (42.9%)	1 (16.7%)	2 (20.0%)
Rectum	3 (13.0%)	2 (28.6%)	0 (0%)	1 (10.0%)
Transverse colon	1 (4.3%)	0 (0%)	0 (0%)	1 (10.0%)
Ileocecal	2 (8.7%)	0 (0%)	0 (0%)	2 (20.0%)
**Histological subtype, *n* (%)**				
Moderately differentiated	13 (56.5%)	5 (71.4%)	3 (50.0%)	5 (50.0%)
Moderately–poorly differentiated	3 (13.0%)	1 (14.3%)	2 (33.3%)	0 (0%)
Poorly differentiated (incl. signet-ring)	4 (17.4%)	0 (0%)	1 (16.7%)	3 (30.0%)
Mucinous	3 (13.0%)	1 (14.3%)	0 (0%)	2 (20.0%)
**PM timing, *n* (%)**				
Synchronous	17 (73.9%)	4 (57.1%)	5 (83.3%)	8 (80.0%)
Metachronous	6 (26.1%)	3 (42.9%)	1 (16.7%)	2 (20.0%)
**Treatment before collection, *n* (%)**				
None	17 (73.9%)	4 (57.1%)	5 (83.3%)	8 (80.0%)
FOLFOX/mFOLFOX	4 (17.4%)	3 (42.9%)	1 (16.7%)	0 (0%)
XELOX	2 (8.7%)	0 (0%)	0 (0%)	2 (20.0%)

PM—peritoneal metastasis; WES—whole-exome sequencing; IHC—immunohistochemistry. Bold type indicates the column and category headings; the sub-items within each category are set in regular type.

## Data Availability

The WES and proteomic data generated in this study are available from the corresponding author upon reasonable request. Publicly available datasets used in this study are available through GEO (GSE132465, GSE183916, GSE225857) and TCGA.

## Supplementary Materials

The following supporting information can be downloaded at: https://www.mdpi.com/article/10.3390/ijms27209106/s1.
