# Supplementary material for: Integrated Multi-Omic Analysis of Paired Primary and Peritoneal Metastatic Tumors Reveals GPAA1 as a Therapeutic Target in Colorectal Cancer
Source: Int J Mol Sci. 2026 Oct 14;27(20):9106. doi: 10.3390/ijms27209106 (PMC13704995; doi:10.3390/ijms27209106)
Supplement: Supplementary file 1 [file ijms-27-09106-s001.zip › ijms-4595130-supplementary.pdf]

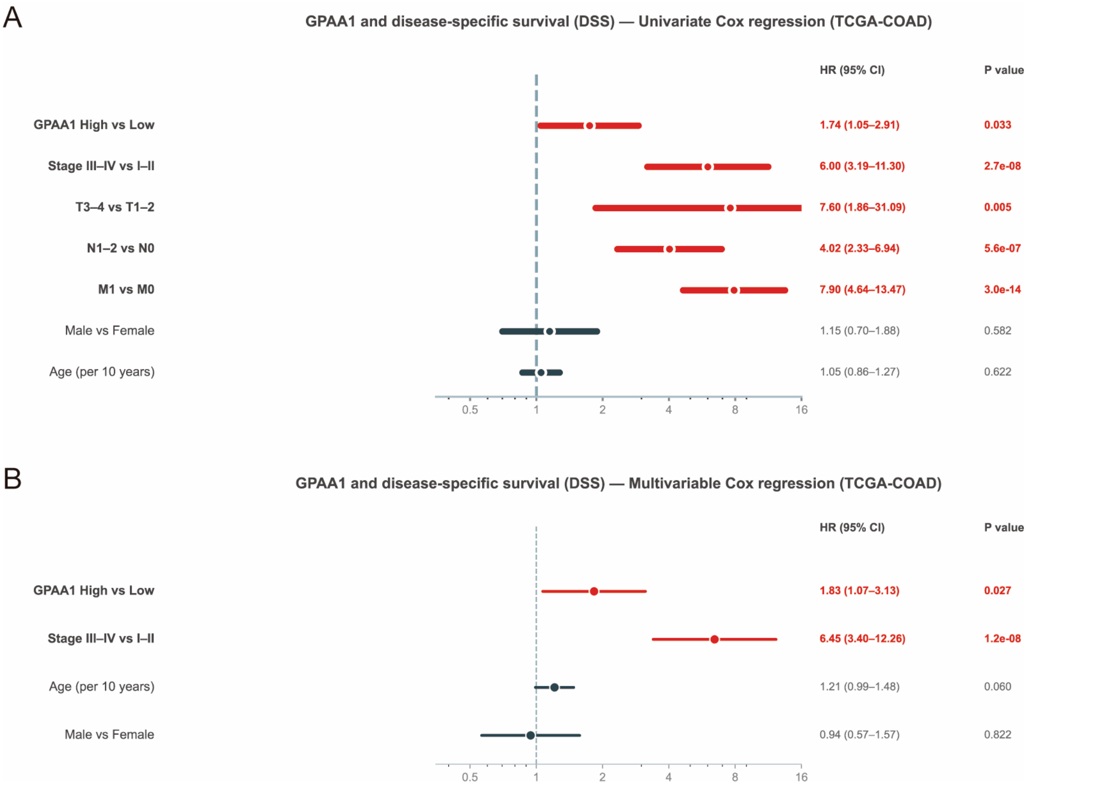

Figure S1. Univariate and multivariable Cox regression analysis of GPAA1 expression and disease-specific survival in colorectal cancer.

(A) Forest plot of univariate Cox proportional hazards regression for disease-specific survival (DSS) in the TCGA-COAD cohort. Hazard ratios (HR) and 95% confidence intervals (95% CI) are shown for GPAA1 expression group (high vs. low, dichotomized at the median), stage (III–IV vs. I–II), T stage (T3–4 vs. T1–2), N stage (N1–2 vs. N0), M stage (M1 vs. M0), sex (male vs. female), and age (per 10 years). Analyses included 439 patients (64 events); stage and M stage data were available for 429 (61 events) and 383 (56 events) patients, respectively. Variables significantly associated with DSS ($P < 0.05$) are highlighted in red; the dashed vertical line indicates HR = 1. The x-axis is on a logarithmic scale.

(B) Forest plot of multivariable Cox proportional hazards regression for DSS in TCGA-COAD, adjusted for GPAA1 expression group (high vs. low), stage (III–IV vs. I–II), age (per 10 years), and sex (n = 429 patients; 61 events; global proportional-hazards test P = 0.627). GPAA1 remained an independent prognostic factor for DSS (HR = 1.83, 95% CI: 1.07–3.13, P = 0.027). DSS, disease-specific survival; HR, hazard ratio; CI, confidence interval.
